# The specificity for the correlation between viscera and somato in chronic stable angina pectoris patients and healthy controls: An assessor-blinded and comparative trial

**DOI:** 10.1371/journal.pone.0331868

**Published:** 2025-09-26

**Authors:** Yongliang Jiang, Xiaoyu Li, Xiaofen He, Hantong Hu, Yajun Zhang, Xiaomei Shao, Jianqiao Fang

**Affiliations:** 1 Department of Neurobiology and Acupuncture Research, The Third Clinical Medical College, Zhejiang Chinese Medical University, Key Laboratory of Acupuncture and Neurology of Zhejiang Province, Hangzhou City, China; 2 Department of Acupuncture and Moxibustion, The Third Affiliated Hospital of Zhejiang Chinese Medical University, Hangzhou City, China; Government Gousia Hospital, DHS, Srinagar, INDIA

## Abstract

**Background:**

Although the relationship between viscera and somato remains unclear, a deeper comprehension of the relationship will maximize the diagnostic and therapeutic benefits. Therefore, this study was conducted to explore the specificity of visceral-somatic associations in the pathological state of chronic stable angina pectoris (CSAP).

**Methods:**

40 individuals with CSAP participated in the study, while 40 individuals in the healthy control group were age-matched healthy individuals. Four distinct somatic locations dispersed along the heart and lung meridians were subjected to laser doppler flowmetry, infrared thermography, and functional near-infrared spectroscopy in order to assess (1) perfusion unit (PU), (2) temperature, and (3) regional oxygen saturation (rSO2). These three outcomes represented the somatic sites’ microcirculatory, thermal, and metabolic properties.

**Results:**

Regarding the microcirculatory characteristics, PU at the somatic sites of the heart meridian (Shenmen(HT7)/Shaohai(HT3)) in the CSAP group substantially increased (*P* < 0.05) compared to the healthy control group, while there was no statistically significant difference in PU at the somatic sites of the lung meridian between the two groups. Regarding the thermal characteristics, compared with the healthy control group, the temperature of the somatic sites of the heart meridian (Shenmen(HT7)/Shaohai(HT3)), as well as Taiyuan (LU9) of the lung meridian, increased significantly (*P* < 0.05). With regard to the metabolic features, rSO_2_ of the somatic site of the heart meridian (Shaohai (HT3)) in the CSAP group decreased significantly (*P* < 0.05) as compared to the healthy control group. The between-group difference in rSO2 in the lung and heart meridians (Taiyuan (LU9)/Chize (LU5)) and Shenmen (HT7), respectively, was not statistically significant.

**Conclusions:**

Specific somatic sites in the heart meridian typically exhibit more significant changes in their microcirculatory, thermal, and metabolic characteristics than those in the lung meridian, thereby supporting the relative specificity for the visceral-somatic association in the disease state of CSAP. **Trial registration**: Clinicaltrials.gov (registration number: NCT04046640)

## 1 Introduction

It is indicated that pathological conditions of visceral organs can cause alterations at certain locations on the somatic sites, including referred somatic hypersensitivity [1]. It frequently results in referred pain that is localized in particular areas of the body’s surface. For instance, pain experienced during a heart attack is frequently felt in the innervated region of the heart on the body surface (e.g., ulnar side of the the upper arm, neck, and thoracic dorsal sites). Moreover, visceral disorders can cause muscle contraction, sympathetic activation, and antidromic activation of afferent fibers by the visceral-somatic reflex, which results in neurogenic inflammation [2]. Additionally, they can cause central sensitization with hypersensitivity and expansion in the number and size of receptive fields, being segmentally predominant at the level of the affected viscera.

The segmental innervation theory is often used to explain such visceral-somatic associations. However, this theory is not able to adequately account for all the facts that represent the visceral-somatic relationship. In actuality, the visceral-somatic association’s principles are extremely complicated, and the underlying processes still remain poorly understood [3]. The diagnostic and therapeutic benefits of the visceral-somatic relationship will be maximized with a greater knowledge of it. For instance, abnormal body temperature at particular locations as determined by contemporary methods is a valuable sign of visceral disorders [4] and its early identification can help in the diagnosis of a variety of diseases, such as liver diseases [5] and tumors [6] and. The growing initiative “Stimulating Peripheral Activity to Relieve Conditions (SPARC)” [7] supported by the National Institutes of Health (NIH) in recent years is a classic illustration of how somatic sites might be stimulated to manage pathological conditions of visceral organs. The SPARC initiative seeks to quicken the creation of therapeutic gadgets that control electrical activity on somatic sites to enhance organ performance [7]. Because of the importance of the visceral-somatic connection, some researchers have proposed the idea of “somatic medicine,” which places an emphasis on the diagnostic and therapeutic benefits of the body’s surface [8].

Similar to this, traditional Chinese medicine (TCM) holds that certain somatic sites, particularly acupoints, can distribute in corresponding meridians and distribute pathological changes of the viscera [9]. In turn, stimulating these involved somatic sites can regulate pathological states of the viscera [10,11]. For instance, cardiovascular diseases can present changes in acupoints in the heart meridian, and in turn, acupoints in the heart meridian can effectively treat cardiovascular diseases [11,12]. Moreover, it is probably relative specific for the relationship between viscera and meridians. For instance, when treating cardiovascular diseases, the therapeutic effect of stimulating somatic sites located in the heart meridian may be superior to sites located in the lung meridian [11]. Nonetheless, the rule of meridian-viscera connection is extremely complex, and its precise underlying processes remain unknown despite a great number of previous research [13]. Therefore, it is urgent to conduct more thorough investigations in this situation.

Taken together, this study selected acupoints along the heart meridian as the primary focus, given their heightened sensitivity in reflecting visceral pathology compared to other body surface sites. The objective is to investigate the specificity of the visceral-somatic relationship under pathological conditions by characterizing multiple biological properties at these sites using three advanced measurement techniques.

## 2 Materials and methods

### 2.1 Study designs

**Fig 1:**
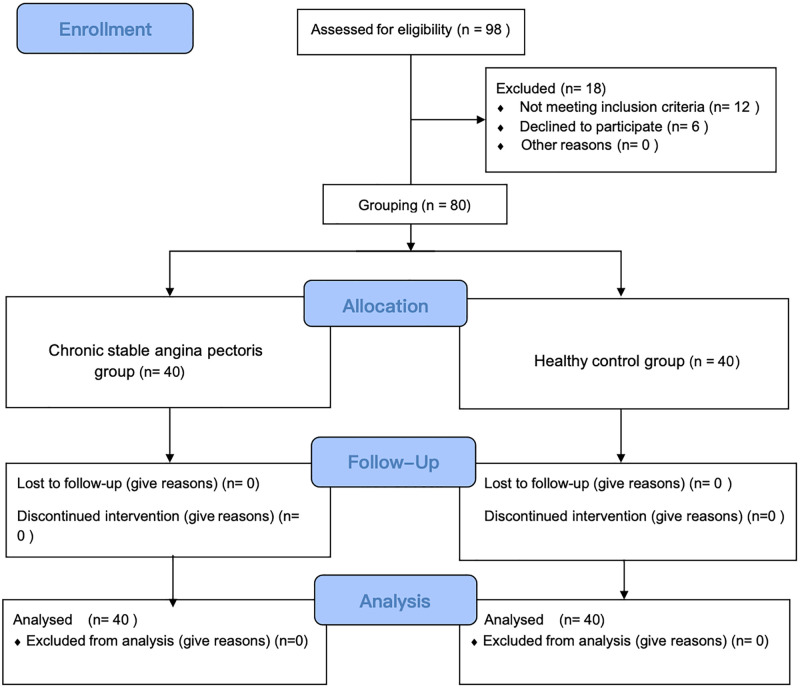
The CONSORT flowchart of the study.

This study was designed as an assessor-blinded comparative trial. The complete date range for participant recruitment and follow-up was from February 1, 2020, to February 1, 2021. Referring to our previously published similar study [14], to comprehensively examine the specificity of visceral-somatic associations, three distinct biological characteristics at somatic sites were assessed in patients with chronic stable angina pectoris (CSAP) and healthy controls using corresponding modern techniques: microcirculatory characteristics via laser Doppler flowmetry (LDF), thermal characteristics via infrared thermography (IRT), and metabolic characteristics via functional near-infrared spectroscopy (fNIRS). The flowchart of the study is presented in Fig 1, illustrating the process of patient inclusion, exclusion, and final analysis. Ultimately, 40 participants were included in the CSAP group, and 40 in the healthy control group. This trial is reported in accordance with the CONSORT 2010 checklist (S1 File), and the detailed study protocol (S2 and S3 Files) and minimal data set (S4 File) are available as Supporting Information files.

### 2.2 Trial registration and ethical approval

This trial has been prospectively registered in Clinicaltrials.gov with the registration number NCT04046640. The Ethics Committee of the Third Affiliated Hospital of Zhejiang Chinese Medical University granted consent (approval No: ZSLL-KY-2019-001A/B/C-01). Each participant completed an informed consent form after receiving complete information about the clinical trial.

### 2.3 Study participants

Patients with CSAP and healthy controls were included as research subjects. According to our earlier study, the aforementioned 3 kinds of biological characteristics of somatic sites between two groups could be influenced by significant age differences, thus, the healthy control volunteers and the CSAP patients had their ages matched in the present study.

#### 2.3.1 CSAP diagnostic standards.

The diagnostic standards for CSAP were based on the “Chronic Angina Focused Update of the ACC/AHA 2002 Guidelines for the Management of Patients With Chronic Stable Angina” [15].

#### 2.3.2 Criteria for inclusion.

(1) Criteria for Including CSAP Patients
  1) Participants were required to meet the diagnostic standards for coronary heart disease, defined by one or more of the following: a) documented history of myocardial infarction, coronary artery bypass graft surgery, or percutaneous coronary intervention; b) angiographic confirmation—via coronary or CT angiography—of ≥50% stenosis in a major coronary artery or critical branch; c) evidence of myocardial ischemia from radionuclide myocardial perfusion imaging during exercise; d) positive treadmill exercise stress testing.
  2) Participants needed to satisfy the CSAP diagnosis and be categorized as Class II or III by the Canadian Cardiovascular Society criteria.
  3) A confirmed CSAP history of over three months was required, with at least two episodes per week during the preceding month.
  4) Eligible participants ranged from 20 to 75 years old, inclusive, irrespective of sex.
  5) Candidates had to be fully conscious and capable of normal communication.
  6) Full comprehension of the research protocol and provision of written informed consent were necessary.
(2) Criteria for healthy controls
  1) Healthy volunteers must have presented a physical examination report, issued within six months before enrollment, confirming their good health.
  2) Age matching with the CSAP cohort was required.
  3) Participants exhibiting dermatological conditions (e.g., pigmentation, redness, swelling, or scarring) at measurement sites on the forearm were excluded.
  4) Participants were required to understand the entire study protocol and consent in writing.

#### 2.3.3 Criteria for exclusion.

(1) For CSAP participants
  1) Individuals with acute coronary syndromes or significant arrhythmias were excluded.
  2) Participants whose chest pain stemmed from valvular disease, dilated or hypertrophic cardiomyopathy were ineligible.
  3) Non-cardiac origins of chest pain were exclusionary.
  4) Those with coexisting pulmonary conditions were not included.
  5) Participants with unmanaged serious comorbidities were excluded.
  6) Subjects with psychiatric disorders, major depression, substance dependence, or a history of drug abuse were not eligible.
  7) Pregnant or lactating individuals were excluded.
  8) Participation in other clinical studies was grounds for exclusion.
(2) For healthy adults.
  1) Individuals who were pregnant or breastfeeding were excluded.
  2) Those with a history of substance or alcohol abuse were not considered.
  3) Concurrent involvement in another research study disqualified candidates.

### 2.4 Sample size determination

This investigation aimed to evaluate and compare biological parameters between CSAP patients and healthy individuals. Since conventional standards for calculating sample sizes are not well-defined for studies of this nature—as opposed to interventional clinical trials—sample size decisions were guided primarily by precedent in related research [16–18] and by logistical considerations in our study’s design. Ultimately, 80 participants were enrolled: 40 diagnosed with CSAP and 40 serving as healthy controls.

### 2.5 Group assignment

Following a rigorous screening process using predefined inclusion and exclusion standards, qualified individuals were placed into either the CSAP cohort or the control group.

### 2.6 Blinding methodology

Given that participants did not undergo any treatment intervention, blinding of subjects was deemed unnecessary. However, outcome assessments were conducted by trained personnel blinded to group allocation. Independent statisticians, with no involvement in the study execution, conducted the final data analyses.

### 2.7 Biological characteristic assessment protocol

Biological characteristic assessment protocol was based on our previously published similar research [14].

#### 2.7.1 Testing conditions.

A dedicated room maintained at 26°C was used for testing. Participants were instructed to avoid vasoreactive medications and agents influencing microcirculation for at least seven days before assessment. They were also required to abstain from smoking and caffeine- or alcohol-containing drinks for 24 hours before testing. Eating and physical activity were restricted for one hour prior. Testing was conducted from 9:00–11:00 AM. Prior to assessments, subjects rested in a supine position for 30 minutes. Baseline physiological indicators—including temperature, heart rate, systolic/diastolic blood pressure, and respiratory rate—were recorded. Throughout measurement, participants maintained steady breathing and avoided limb movement. Female subjects were scheduled outside the menstrual phase to avoid thermal interference from basal body temperature.

#### 2.7.2 Body surface sites of measurement.

**Fig 2:**
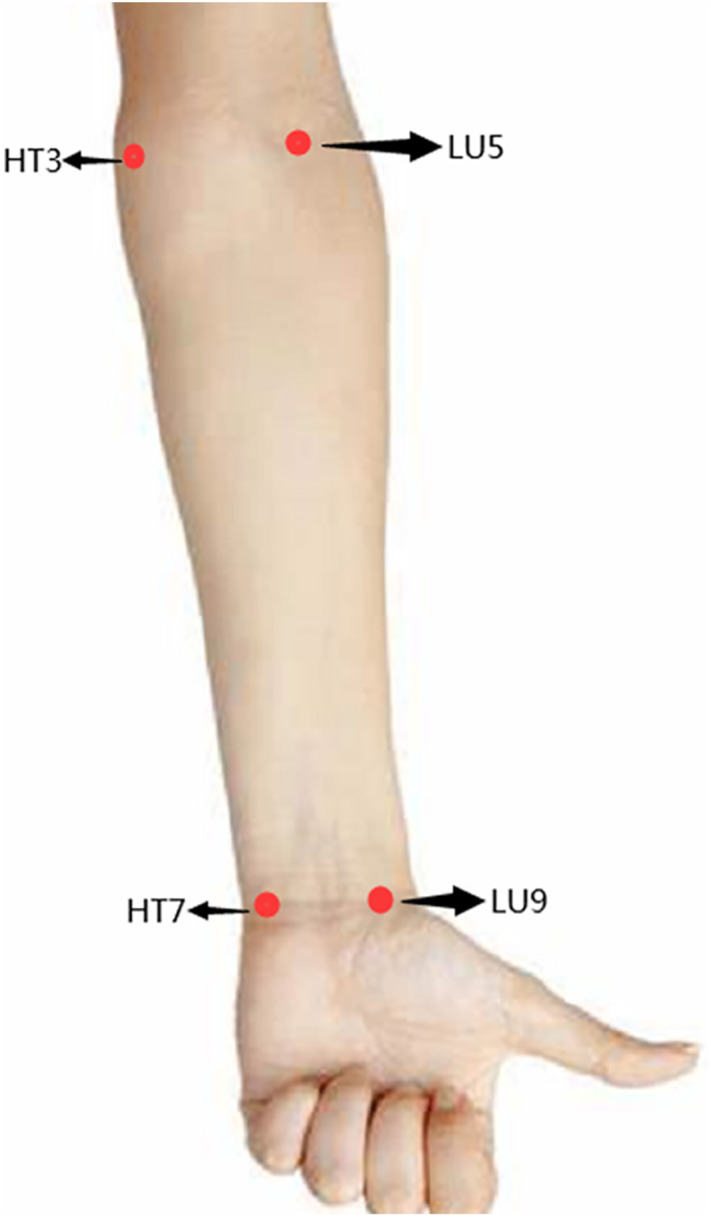
The schematic diagram for 4 somatic measurement sites.

As illustrated in Fig 2, four anatomical locations were selected: Shenmen (HT7), Shaohai (HT3), Taiyuan (LU9), and Chize (LU5). Table 1 details their anatomical references.

**Table 1:** Location of 4 somatic measurement sites.

Measurement sites	Location
Shenmen (HT7)	On the anteromedial aspect of the wrist, radial to the flexor carpi ulnaris tendon, on the palmar wrist crease.
Shaohai (HT3)	On the anteromedial aspect of the elbow, just anterior to the medial epicondyle of the humerus, at the same level as the cubital crease.
Taiyuan (LU9)	On the anterolateral aspect of the wrist, between the radial styloid process and the scaphoid bone, in the depression ulnar to the abductor pollicis longus tendon.
Chize (LU5)	On the anterior aspect of the elbow, at the cubital crease, in the depression lateral to the biceps brachii tendon.

#### 2.7.3 Procedures of examination.

(1) Laser Doppler Flowmetry (LDF)

**Fig 3:**
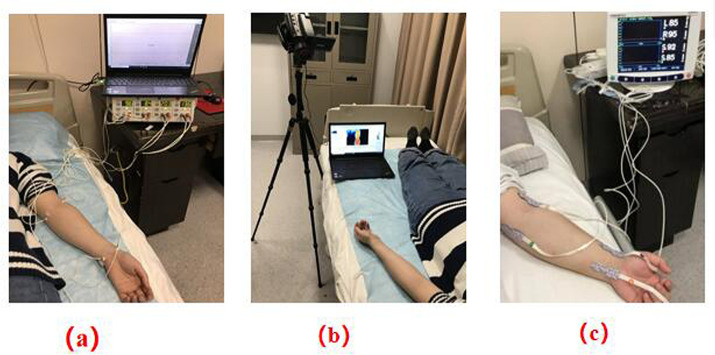
The schematic of the experiment. **(a)** LDF detection. **(b)** IRT detection. **(c)** fNIRS detection.

Microcirculatory perfusion was assessed with a four-channel LDF system (PeriFlux System 5000, Sweden). Measurements were recorded in perfusion units (PU) using Perisoft for Windows. Sensors (Probe 407) were affixed at the four selected acupoints, and PU values were continuously logged for 10 minutes. Fig 3(a) shows the LDF setup.

(2) Infrared Thermography (IRT)

Thermal imaging of the skin was captured every minute during a 10-minute session using an infrared thermograph (NEC InfRec R450, Avio, Tokyo). Images were processed with InfRec Analyzer NS9500 software. Temperatures at each site were measured and averaged. A visual of the IRT protocol is given in Fig 3(b).

(3) Functional Near-Infrared Spectroscopy (fNIRS)

Metabolic activity was measured via a four-channel oximeter (INVOS 5100C, Somanetics, USA). Probes (OxyAlert™) were placed at the same somatic sites to record oxygen saturation over 10 minutes. Regional oxygen saturation (rSO2) was derived using INVOS Analytics software. Fig 3(c) presents the schematic for this procedure.

### 2.8 Outcomes assessed

(1) Microcirculatory characteristics: PU values.
(2) Thermal characteristics: infrared thermographic imaging, average acupoint temperature, and mean meridian temperature.
(3) Metabolic characteristics: regional oxygen saturation (rSO2).

### 2.9 Statistical analysis

Statistical analysis was conducted using SPSS version 25.0 for Windows (SPSS Inc., Chicago, IL, USA). Initially, all datasets were assessed for normal distribution. When data followed a normal distribution, continuous variables were presented as mean ± standard error of the mean (SEM). In such cases, within-group comparisons were performed using paired t-tests, and between-group differences were analyzed with independent samples t-tests. For non-normally distributed variables, data were summarized as medians with interquartile ranges (M [Q1, Q3]). Comparisons between independent groups were carried out using the Mann–Whitney U test, while within-group differences were evaluated with the Wilcoxon signed-rank test. A two-tailed P-value of less than 0.05 was considered statistically significant.

## 3 Results

40 individuals (23 men and 17 women, ages ranging from 39 to 75 years old) were enrolled in the CSAP group. The healthy control group consisted of 40 healthy participants (20 men and 20 females, ages ranging from 49 to 75 years). Between the two groups, there was no discernible difference in age or gender (P > 0.05).

### 3.1 Comparison of microcirculatory characteristics

**Fig 4:**
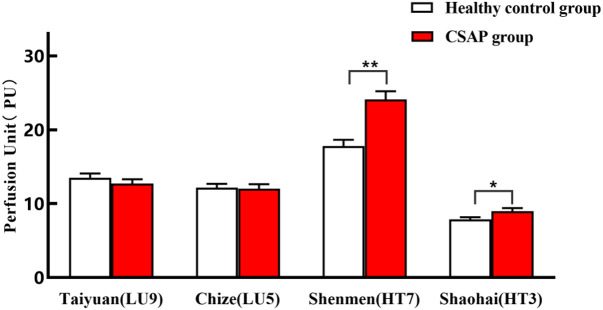
Comparison of PU on specific acupoints of heart and lung meridians between the two groups. P* < 0.05 and **P < 0.01 compared with the healthy control group.

According to Table 2 and Fig 4, the PU of the Shenmen(HT7)/Shaohai(HT3) of the heart meridian significantly increased in the CSAP group as compared to the healthy control group (P < 0.01). The Yuan-Primary point of the heart meridian, Shenmen(HT7), showed the largest growth, with a mean increase of 6.32 units. However, there was no statistically significant difference in PU in Taiyuan(LU9)/Chize(LU5) between the two groups (P > 0.05).

**Table 2:** Comparison of PU on specific acupoints of heart and lung meridians between two groups (mean ± SEM).

Group	Lung meridian		Heart meridian	
	Taiyuan(LU9)	Chize(LU5)	Shenmen(HT7)	Shaohai(HT3)
Healthy control group	13.51 ± 0.57	12.17 ± 0.50	17.77 ± 0.87	7.86 ± 0.32
CSAP group	12.72 ± 0.57	12.00 ± 0.63	24.09 ± 1.10**	8.97 ± 0.44*

P* < 0.05 and ^**^P < 0.01 compared with the healthy control group.

### 3.2 Comparison of thermal characteristics

(1) Comparison of infrared thermal images

**Fig 5:**
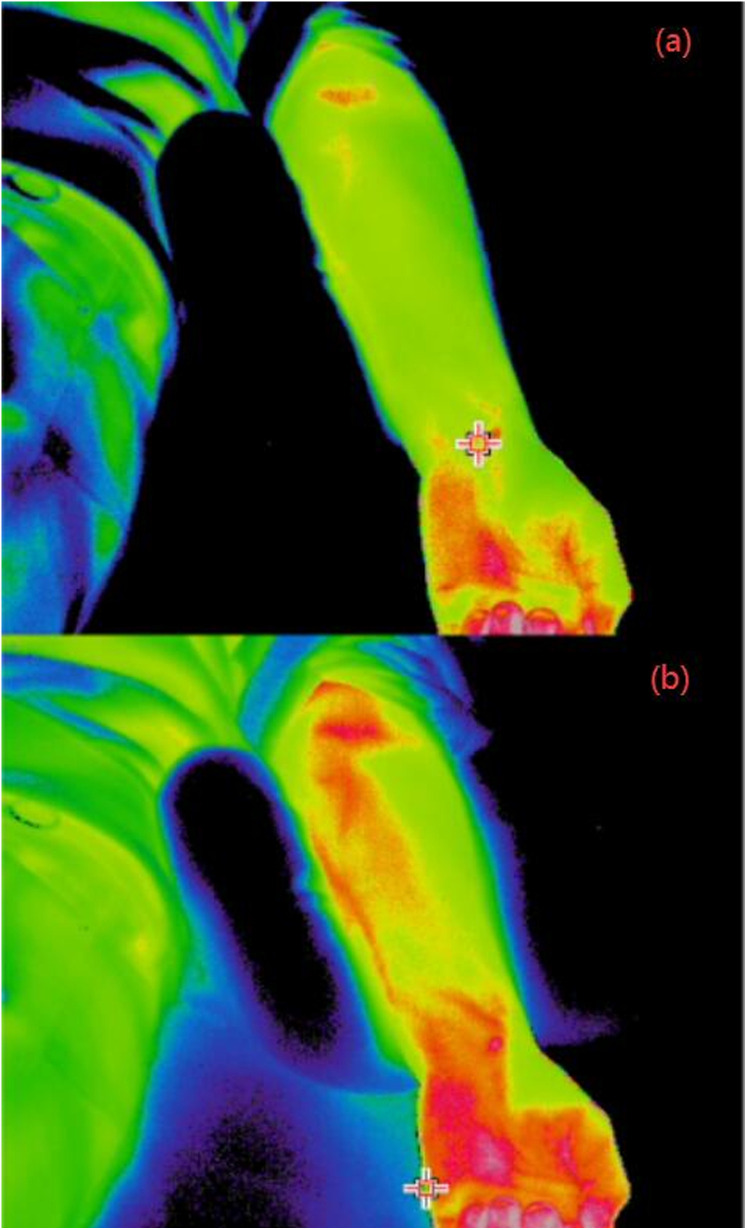
Infrared thermal images of typical subjects. **(a)** Healthy control group; **(b)** CSAP group.

As shown in Fig 5(a), the heart and lung meridians’ circulation areas in the healthy control group’s forearm displayed moderate temperatures on the infrared thermal pictures of. In the circulation zones of both meridians, there were no abnormally high temperature spots or strip-like high temperature regions.

As seen in Fig 5(b), significant hyperthermia areas were seen in the circulation regions of the heart meridian (located in the ulnar side of the medial forearm) among some CSAP patients, representing as red dots, sheets, or strips. In comparison to healthy controls; however, no abnormal hyperthermia areas were found in the circulation regions of the lung meridian circulation (located in the radial side of the medial forearm).

(2) Comparison of mean acupoint temperature

**Fig 6:**
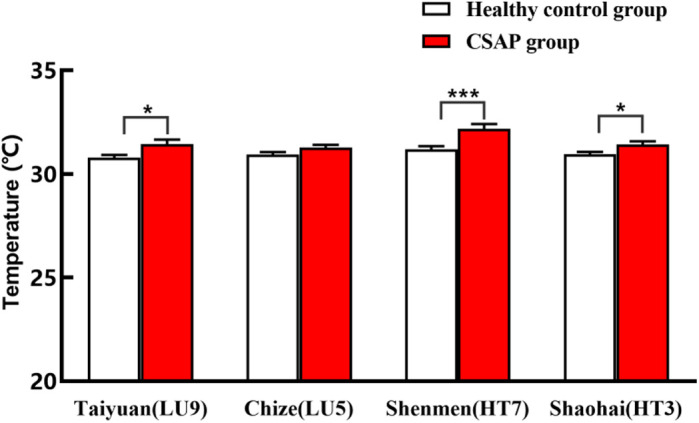
Comparison of mean temperature on specific sites of heart and lung meridians between the two groups. ***P < 0.001, **P < 0.01, and *P < 0.05 compared with the healthy control group.

As seen in Table 3 and Fig 6, the temperature of Shenmen (HT7)/Shaohai (HT3) in the heart meridian, as well as Taiyuan (LU9) of the lung meridian, increased considerably (P < 0.05) in the CSAP group compared to the healthy control group. However, there was no statistically significant difference in the lung meridian’s Chize (LU5) temperature between the two groups (P > 0.05).

**Table 3:** Comparison of mean temperature on specific acupoints of heart and lung meridians between two groups (mean ± SEM).

Group	Lung meridian		Heart meridian	
	Taiyuan(LU9)	Chize(LU5)	Shenmen(HT7)	Shaohai(HT3)
Healthy control group	30.78 ± 0.14	30.93 ± 0.12	31.18 ± 0.16	30.96 ± 0.11
CSAP group	31.44 ± 0.21*	31.27 ± 0.13	32.18 ± 0.23***	31.42 ± 0.15*

*** *P* < 0.001, ** *P* < 0.01, and * *P* < 0.05 compared with the healthy control group

### 3.3 Comparison of metabolic characteristics

**Fig 7:**
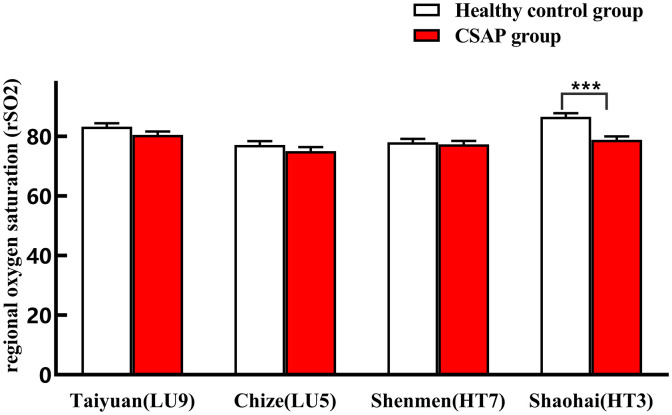
Comparison of rSO_2_ on specific acupoints of heart and lung meridians between the two groups. **P < 0.01 compared with the healthy control group.

Table 4 and Fig 7 illustrate the lowering trend in rSO2 at 4 somatic sites of the lung meridian and lung meridian in the CSAP group. The rSO2 of Shaohai (HT3) of the heart meridian considerably decreased in the CSAP group when compared to the healthy control group (P < 0.05). However, the between-group difference of rSO2 in Shenmen (HT7) of the heart meridian, as well as Taiyuan(LU9)/Chize(LU5) of the lung meridian was not statistically significant (P > 0.05).

**Table 4:** Comparison of rSO_2_ on specific acupoints of heart and lung meridians between two groups (mean  $\bar{x}$$\bar{x}$$\bar{x}$. ± SEM).

Group	Lung meridian		Heart meridian	
	Taiyuan(LU9)	Chize(LU5)	Shenmen(HT7)	Shaohai(HT3)
Healthy control group	83.29 ± 1.14	77.16 ± 1.26	78.06 ± 1.14	86.58 ± 1.26
CSAP group	80.54 ± 1.16	75.06 ± 1.39	77.36 ± 1.18	78.84 ± 1.17***

***P < 0.001 compared with the healthy control group

## 4 Discussions

### 4.1 Primary findings and potential mechanisms

In terms of the microcirculatory and thermal characteristics of the body surface during the pathological state of the heart, we discovered that the PU and temperature of specific somatic sites (e.g., Shenmen (HT7)/Shaohai (HT3)) in the heart meridian were significantly higher compared to healthy controls, whereas the PU and temperature of other somatic sites in the lung meridian (e.g., Taiyuan (LU9)/Chize (LU5)) did not change significantly. With regard to metabolic characteristics, we discovered that rSO_2_ in specific somatic sites of the heart meridian (i.e., Shaohai (HT3)) were significantly lower than those of healthy controls.

Taken together, we can conclude that specific somatic sites in the heart meridian typically exhibit more significant changes in their microcirculatory, thermal, and metabolic characteristics than those in the lung meridian. Therefore, there is relative specificity for the visceral-somatic association in the pathological state of CSAP.

Similar findings have been observed in other diseases. For example, a study [19] discovered that individuals with lung disorders had considerably higher temperatures at certain distant acupoints in the lung meridian compared with the heart meridian and the pericardium meridian

In addition, regarding the change trend of PU, temperature and rSO2, PU and temperature in specific somatic sites (Shenmen (HT7)/Shaohai (HT3) in the heart meridian) in the superficial layer of the skin increased significantly for CSAP patients in our study, whereas rSO_2_ in specific somatic sites (Shenmen (HT7)/Shaohai (HT3) in the heart meridian) decreased significantly, several factors may be invloved. From a TCM perspective, Shenmen (HT7) and Shaohai (HT3) are crucial acupoints on the Heart Meridian; Shenmen (HT7) is the Yuan-Source acupoint, and Shaohai (HT3) is the He-Sea acupoint. Yuan-Source and He-Sea acupoints are traditionally considered important for reflecting and treating disorders of their related Zang-Fu organs. The observed increases in PU and temperature at these sites in CSAP patients may be indicative of acupoint sensitization, a phenomenon often accompanied by heightened microcirculatory perfusion and superficial temperature based on previous experimental research [20]. This sensitization could be a somatic manifestation of the underlying cardiac pathology in CSAP. Conversely, the significant decrease in rSO2 at Shaohai (HT3) might reflect the chronic ischemic and hypoxic state characteristic of CSAP [21,22], leading to reduced oxygen saturation in the deeper tissues at this specific acupoint. The convergence of these multi-modal changes at Shenmen (HT7) and Shaohai (HT3) underscores their potential importance in the context of CSAP and warrants further investigation into the precise mechanisms integrating TCM theory with physiological manifestations.

### 4.2 Strengths and novelties of our research

It is noteworthy that our study contains the following strengths and novelties. First, while many earlier research sought to investigate the relationship between meridians and viscerae, the vast majority of them concentrated on the relationship between a single meridian and a single viscus [23]. However, the rules for meridian-viscera connection are intricate, and the precise mechanisms that underlie it are still unknown [24,25]. While numerous previous studies have explored meridian-viscera relationships, this study offers a novel approach by not only focusing on the Heart Meridian in relation to CSAP but also by directly comparing these findings with the Lung Meridian simultaneously, using multiple modern techniques. To the best of our knowledge, there are also very few studies that specifically address the uniqueness of the relationship between various meridians and various viscera, particularly concerning whether specific somatic locations on the Heart Meridian are uniquely sensitive compared to other meridians or non-meridian sites in manifesting aberrant biological traits. As a result, the findings of the current study will help clarify the principles governing the specificity for meridian-viscera connection in TCM and the visceral-somatic association in contemporary medicine, supporting their diagnostic and therapeutic merits.

Second, another element of our study’s novelty is that the majority of earlier studies in this field focused on the biological characteristics of measurement sites in the reaction regions of the viscus distributing on the back or the localized sites of the impaired viscus, whereas the measurement sites of interest in the current study were distal somatic sites away from the viscus to primarily investigate whether there is relative specificity for the somato-viscera association in pathological states. Our results demonstrated that specific somatic sites in the heart meridian, which is directly related to the heart in TCM, had biological characteristics that changed significantly in the disease state of the CSAP, whereas the lung meridian, which is not directly related to the heart, had biological characteristics that generally did not change significantly. Therefore, our study results support the relative specificity for somato-viscera interaction in particular areas during physiological states and provide some insight for contemporary research, including the upcoming SPARC program [26,27].

Third, to the best of our knowledge, this is the first study to simultaneously utilize three kinds of techniques (LDF, IRT, fNIRS) to examine a variety of biological traits at somatic sites, including the microcirculatory, thermal, metabolic, and electrical traits of the body surface. This multi-modal approach provides a more comprehensive insight into the physiological changes occurring at these specific body surface locations, which represents a methodological advancement in this area of research. This multi-modal approach provides a more comprehensive insight into the physiological changes occurring at these specific body surface locations, which represents a methodological advancement in this area of research. Due to their benefits of non-invasiveness, fast response, ease, tissue specificity, and repeatability, these three techniques have generally been utilized extensively in research in this field [28–30], demonstrating their accuracy and dependability.

Fourth, we used a variety of strategies to minimize the confounding variables that could have an impact on the biological characteristics. These strategies included controlling variables related to the environment, variables related to physiological changes, and variables related to drugs and diet.

### 4.3 Clinical significance of our study

The findings of this study, which support the relative specificity of the visceral-somatic association in CSAP, hold potential clinical significance. The ability to detect specific changes in microcirculatory, thermal, and metabolic characteristics at particular Heart Meridian acupoints could contribute to non-invasive diagnostic approaches for CSAP. For instance, abnormal body temperature at particular somatic sites can be a valuable sign of visceral disorders, aiding in early diagnosis. Furthermore, a deeper understanding of these specific meridian-viscera links could inform the development of more targeted therapeutic interventions, potentially guiding initiatives similar to the SPARC program [26,27], which aims to modulate peripheral nerve activity for therapeutic benefit. Clarifying these principles supports both TCM diagnostic/therapeutic merits and contemporary medical understanding

### 4.4 Limitations

The limitations of our investigation should be acknowledged. Frist, the sample size is firstly somewhat small. While the study had 40 participants in each group, future research with larger cohorts would be beneficial to enhance statistical power and the generalizability of the findings. However, the outcomes of this pilot study can serve as a guide for determining if the trial approach is feasible and can also produce preliminary data that will help future large-scale investigations. Second, the data were collected between February 2020 and February 2021. While these findings provide valuable insights from that period, future studies could aim to incorporate more recent data to account for any potential temporal variations or evolving factors. Third, only 4 measurement sites on the body surface were concurrently identified due to the current degree of method development, where the device of LDF and fNIRS can only be equipped with a maximum of 4 probes. We did not measure the biological properties of locations found on non-meridians or non-acupoints found in meridians. Future research should evaluate more somatic locations using more sophisticated tools to more thoroughly and methodically study the specificity for the visceral-somatic connection.

## 5 Conclusions

In the disease state of CSAP, specific somatic sites in the heart meridian typically exhibit more significant changes in their microcirculatory, thermal, and metabolic characteristics than those in the lung meridian. Therefore, there is relative specificity for the visceral-somatic association in the pathological state of CSAP.

## Supporting information

S1 File CONSORT-2010-Checklist. (DOC)

S2 File Study protocol (Chinese version). (DOC)

S3 File Study protocol (English Translation Version). (DOCX)

S4 File Minimal data set. (XLSX)

## Abbreviations

SPARC: stimulating peripheral activity to relieve conditions
NIH: National Institutes of Health
TCM: traditional Chinese medicine
LDF: laser doppler flowmetry
IRT: infrared thermography
fNIRS: functional near-infrared spectroscopy
CSAP: chronic stable angina pectoris
PU: perfusion units
rSO_2_: regional oxygen saturation.
