# Supplementary material for: The specificity for the correlation between viscera and somato in chronic stable angina pectoris patients and healthy controls: An assessor-blinded and comparative trial
Source: PLoS One. 2025 Sep 26;20(9):e0331868. doi: 10.1371/journal.pone.0331868 (PMC12469238; doi:10.1371/journal.pone.0331868)
Supplement: S2 File — (DOC) [file pone.0331868.s002.doc]

**临床试验研究方案**

**项目名称** 心、肺二经循经经脉现象的生物学特征

**负 责 人**  方剑乔

**组长单位:** 浙江中医药大学

# 目录

[**目录** 1](#__RefHeading___Toc333352870)

[**研究方案** 2](#__RefHeading___Toc333352871)

[**一、研究背景** 2](#__RefHeading___Toc333352872)

[**二、研究目的** 3](#__RefHeading___Toc333352873)

[**三、研究计划** 3](#__RefHeading___Toc333352874)

[1. 受试者选择标准 3](#__RefHeading___Toc333352875)

[2．受试者招募人数 6](#__RefHeading___Toc333352876)

[3．研究者选择来源 6](#__RefHeading___Toc333352877)

[4．研究时间 6](#__RefHeading___Toc333352878)

[5．研究的执行 6](#__RefHeading___Toc333352879)

[6．观察指标 8](#__RefHeading___Toc333352879)

[**四、不良事件的处理** 9](#__RefHeading___Toc333352880)

[1．不良事件的类型 9](#__RefHeading___Toc333352881)

[2．严重性 9](#__RefHeading___Toc333352882)

[3．不良事件的识别、记录和处理 9](#__RefHeading___Toc333352883)

[4．严重不良事件的报告 9](#__RefHeading___Toc333352883)

[**五、伦理学和质量** 9](#__RefHeading___Toc333352884)

[**六、数据管理** 9](#__RefHeading___Toc333352885)

[**七、统计分析** 1](#__RefHeading___Toc333352886)0

[1．统计软件 1](#__RefHeading___Toc333352887)0

[2．数据描述 1](#__RefHeading___Toc333352888)0

[3．数据统计 1](#__RefHeading___Toc333352889)0

[4．统计分析计划 1](#__RefHeading___Toc333352890)0

[**八、最终报告和发表** 1](#__RefHeading___Toc333352891)0

[**九、质量控制** 1](#__RefHeading___Toc333352892)0

[十**、参考文献** 1](#__RefHeading___Toc333352891)1

[**十一、附件** 1](#__RefHeading___Toc333352892)2

# 研究方案

### 一、研究背景

## 经络理论是中医基础理论的核心内容之一，指导着包括针灸在内的几乎所有的中医临床实践。经络理论研究无论在理论上还是在应用上都具有十分重要的意义。

## 我国经络研究起源于五六十年代，主要探索经络实质；七十年代循经感传的研究掀起经络研究的高潮，从组织特性的寻找转向循经传导特性的观测。国内多家研究机构率先对循经感传展开调查，初步肯定了经络现象的客观存在。八十年代中期转向应用声、光、电、热等物理手段检测脉络，进一步明确了经脉循行线的理化特性。九十年代至今进入了多角度、多层次的研究阶段，发现经脉线上Ca^2+^等多种离子、神经递质等均高于非经脉线；经脉与脏腑存在特异性的联系。伴随着经络研究的进展，提出了许多经络假说，如神经体液调节假说、血管淋巴管假说、二重反射假说、第三平衡系统假说和低流阻通道假说等，但这些假说有待进一步完善和证实，距离真正阐明经络实质还相距甚远。

## 国外同行在经络研究方面也取得了一定成果，如日本在循经感传方面开展了大量研究；韩国尝试探索经络实质，提出了原始管道系统概念；法国最早使用红外热像技术研究经络，应用磁探测技术揭示了经脉的一些磁学特性；前苏联应用偏光检测技术研究经脉光学特性；英国应用磁探测电阻抗成像技术创造性地对经络进行三维成像。

## 纵观国内外经络研究的现状，虽然取得了重要的进展，但尚未形成重大突破。经络实质仍未明确，研究成果尚不足以指导针灸临床。经络研究存在结构(物质基础)研究与功能研究脱节、基础研究与临床脱节等问题，循经感传等经脉现象的研究结果包含较多主观性的成分，缺少科学的技术和手段呈现经脉现象并探讨其生物学特征的研究。经脉脏腑相关研究更多的是经穴脏腑相关的研究，并不能很好地反映经脉与脏腑的联系及其规律；且主要围绕一经一脏的对应关系，缺少多经多脏间相关性的比较研究，缺少两经及以上同一横断面效应的比较。因此，经络研究需要回归经络理论本源，在研究思路、设计和研究方法上提升创新，对经脉理论尤其是经脉系统循经反应特征及机制、经脉不同部位间特定联系现象、规律及其机制进行研究，将能更好地阐明经络理论的关键共性问题，完善经络理论，并更好地为中医学尤其是针灸临床服务。

基于以上，本项目聚焦于“经络体现的是躯体不同部位之间的联系”这一核心，以心、肺二经的经脉循行为切入点，借助激光多普勒、红外热成像及近红外光谱这三种现代科学技术，检测并探索经脉循经反应所呈现的生物学特征。同时对心肺二经的特异性进行研究，对经络所能反映的躯体不同部位之间的联系进行探索，阐明其联系特点及规律。本研究的结果将对经络学说中至今在临床仍具有重大价值的经验事实与规律给予新的科学的解释，而且对当今经络理论乃至针灸理论的重构以及针灸学的发展都具有重要意义。

## 二、研究目的

(1) 探索生理、病理状态下健康受试者和心肺疾病患者的循经经脉现象生物学特征的差异，同时探索心肺二经与心肺二脏联系的相对特异性。

(2) 建立心肺二经循经经脉现象生物学特征的规范检测技术与方案，为经络理论和针灸理论的重构奠定科学基础。

## 三、研究计划

### 1. 受试者选择标准

本研究的受试者包括以下2种人群：冠心病稳定型心绞痛(stable angina pectoris，SAP)患者以及健康受试者。

### 诊断标准

**1.1.1 SAP诊断标准**

参照美国心脏病学院/美国心脏学会(ACC/AHA)2007年联合颁布的《2007 Chronic Angina Focused Update of the ACC/AHA 2002 Guidelines for the Management of Patients With Chronic Stable Angina》^[1]^ 和中华医学会心血管病学分会2007年颁布的《慢性稳定性心绞痛诊断与治疗指南》^[2]^中关于冠心病稳定型心绞痛的诊断标准：

1. 疼痛部位：典型的心绞痛部位是在胸骨后或左前胸，范围常不局限，可以放射到颈部、咽部、颌部、上腹部、肩背部、左臂及左手指内侧，也可以放射至其他部位，心绞痛还可以发生在胸部以外，如上腹部、咽部、颈部等。每次心绞痛发作部位往往是相似的；
2. 疼痛性质：常呈紧缩感、绞榨感、压迫感、烧灼感、胸憋、胸闷或有窒息感、沉重感，有的患者只述为胸部不适，主观感觉个体差异较大，但一般不会是针刺样疼痛；
3. 持续时间：呈阵发性发作，持续数分钟，一般不会超过10分钟，也不会转瞬即逝或持续数小时；
4. 诱发因素及缓解方式：慢性稳定性心绞痛的发作与劳力或情绪激动有关，如走快路、爬坡时诱发，停下休息即可缓解，多发生在劳力当时而不是之后。舌下含服硝酸甘油可在2-5分钟内迅速缓解症状；
5. 心绞痛发作的程度、频度、性质及诱发因素在数周内无显著变化；
6. 其它检查的支持诊断，如实验室检查、心电图检查、超声心动图、冠脉动脉造影等。

### 1.2 纳入标准

**1.2.1 SAP患者纳入标准**

(1) 符合西医冠心病诊断标准(符合以下任意一项)：①明确的陈旧性心梗病史，或有经皮冠状动脉支架植入术(PCI)史，或冠脉搭桥史者；②冠脉造影或冠脉CTA结果提示至少一支冠脉狭窄且管腔狭窄≥50%者；③负荷核素心肌扫描检查提示冠心病心肌缺血者；④运动平板心电图阳性(限男性患者)。

(2) 符合西医稳定性心绞痛诊断标准，且满足加拿大心血管学会(CCS)心绞痛严重程度分级II-III级的稳定型心绞痛患者(相关分级标准详见附件1)；

(3) 有3个月及以上的心绞痛病史，且近1月内每周有不少于2次发作；

(4) 20岁≤年龄≤75岁，性别不限；

(5) 意识清楚，能完成正常交流者；

(6) 理解并愿意服从研究方案，同时签署知情同意书者。

注：同时符合以上六项的患者，方可纳入本项研究。

**1.2.2 健康志愿者纳入标准**

(1) 能提供近三月内的体检报告并由研究者进行常规体格检查，确认无心脏及肺脏疾病，无消化、泌尿、血液、内分泌、神经系统等严重基础疾病的健康受试者；

(2) 年龄与SAP组受试者匹配，性别不限；

(3) 意识清楚，能完成正常交流者；

(4) 理解并愿意服从研究方案，同时签署知情同意书者。

注：同时符合以上四项的患者，方可纳入本项研究。

**1.3 排除标准**

**1.3.1 SAP排除标准**

(1) 急性冠脉综合征(包括急性心肌梗死和不稳定型心绞痛)、严重心律失常(如重度房室传导阻滞、室性心动过速、影响血流动力学的室上性心动过速、频发早搏特别是室性早搏)的患者；

(2) 瓣膜性心脏病、肥厚型心肌病、扩张型心肌病等引起的胸痛患者；

(3) 非心脏性疾病引起的胸痛患者(如重度神经官能症、更年期综合征、颈椎病、食管/肺部/胸壁病变引起的胸痛)；

(4) 合并肺部疾病的患者；

(5) 合并消化、泌尿、血液、神经等系统的严重基础疾病且目前未能有效控制病情者；

(6) 精神病患者、严重抑郁症患者、酒精依赖者或有药物滥用史者；

(7) 妊娠或哺乳期患者；

(8) 目前正在参加其他临床试验的患者；

注：凡符合上述任一项者， 均予以排除。

**1.3.2 健康志愿者排除标准**

(1) 研究期间突发心脑血管、呼吸、肝、肾、泌尿、造血系统等重大疾病的受试者；

(2) 精神病患者、严重抑郁症患者、酒精依赖者或有药物滥用史者；

(3) 处于妊娠或哺乳期的受试者；

(4) 目前正在参加其他临床试验的受试者；

注：凡符合上述任一项者，均予以排除。

### 1.4 剔除和脱落标准

(1) 不符合纳入标准而被误纳入的病例；

(2) 入组后受试者依从性差，不能遵循试验要求，不配合检测者；

(3) 研究期间因其它个人原因而自行退出者；

(4) 研究期间出现病情急性加重或其他急危重症，需采取抢救措施或住院治疗者；

(5) 发生严重不良事件或者并发症，不能继续接受研究而被中止试验者。

**1.5 剔除和脱落病例的处理**

当受试者脱落后，研究者应采取登门或电话等方式，尽可能与受试者取得联系并记录脱落原因，记录最后一次检测时间，保留所有已完成的检测数据。因不良反应或病情急性加重而退出试验的病例，研究者应根据受试者实际情况采取相应的治疗措施。所有剔除和脱落患者的原始病历应保留备查。

### 2．受试者招募人数

本临床试验预计纳入80名受试者，分别为40位SAP患者以及40位健康受试者。

1. **受试者来源**

SAP患者主要从浙江中医药大学附属第三医院的相关门诊和病房进行招募。健康受试者主要从浙江中医药大学附属第三医院的体检中心进行招募。

1. **研究起止时间**

预计为2020年1月-2021年1月

1. **研究的执行**

**5.1 样本量估算**

本项目为采用多种现代科学技术检测偱经经脉现象所呈现的生物学特征的观察研究，与普通临床试验相比，样本量估算上尚无统一的标准。参考国内外类似的经络生物学特性检测试验^[4-6]^，同时根据本研究的实际条件，拟纳入2组受试者。其中SAP组40例患者，健康组40例志愿者，合计80例受试者。

### 5.2 受试者入选

在开始研究前，研究者应清楚、口语化地向受试者或其授权人详细解释本研究的目的、内容及其潜在风险和益处，受试者或其授权人和研究者在知情同意书上签名和注明日期。受试者或其授权人只有在签署了知情同意书后才能进入筛选并继而参加本研究。

签署书面知情同意书的受试者需要接受以下评估，并进行记录，同时根据纳入与排除标准，判断受试者能否纳入本研究。

1. 人口学及疾病资料：人口学资料包括受试者的性别、姓名、年龄、职业、身高、体重等，疾病资料包括疾病诊断、现病史、既往史、疾病分级及目前服用药物情况等。
2. 生命体征：包括体温、心率、呼吸、血压；
3. 实验室检查：血常规、生化常规（包括AST、ALT、BUN、Cr、GLU、血脂四项等）；
4. 心电图检查。

### 5.3 分组

将80名受试者分别纳入SAP组及健康组，每组各40例。

### 5.4 盲法

本研究实施过程中，对受试者、指标检测与录入者均不设盲。资料总结阶段采用盲法统计分析，由不参与前期试验的第三方统计者进行后期的数据统计分析。

### 5.5 检测方案

分别采用激光多普勒、红外热成像、近红外光谱技术检测3组受试者在生理、病理状态心肺二经的微循环特性、热传输特性和代谢特性。

**5.5.1 穴位定位**

穴位定位参照《腧穴名称与定位》国家标准(GB/T 12346-2006)。

太渊(LU9)：位于腕前区，桡骨茎突与舟状骨之间，拇长展肌腱尺侧凹陷中。

尺泽(LU5)：位于肘横纹中，肱二头肌腱桡侧凹陷处。

神门(HT7)：位于腕前区，腕掌侧横纹尺侧端，尺侧腕屈肌腱的桡侧凹陷处。

少海(HT3)：位于肘前区，横平肘横纹，肱骨内上髁前缘。

**5.5.2 检测注意事项**

(1) 在整个研究周期里，SAP组的患者保持当前的治疗方案不变。期间若因各种原因加用了新的药物或其它治疗手段，须及时向研究者说明，由研究者进行详细记录。健康组的受试组保证研究期间不服用任何药物，若突发疾病使用了药物和其它治疗措施，同样由研究者进行记录，并评估是否予以剔除。

(2) 嘱咐所有受试者在检测的前两天内禁止饮茶、饮酒及喝咖啡，禁止抽烟。检测前1小时内禁止运动和摄入任何食物。

(3) 嘱咐受试者在整个检测过程中持续保持安静，保持自然呼吸，尽量避免肢体的移动。

**5.5.3 检测环境**

设置专门的检测室，室温控制在(25±1)℃，相对湿度控制在40%-50%。室内无阳光直射和明显空气对流。

**5.5.4 微循环特性检测**

(1) 检测设备：PeriFlux System 5000型号四通道激光多普勒血流仪(瑞典PeriMed公司产)

(2) 检测过程：受试者进入检测室，仰卧休息15 min，随后开始正式检测。将4个探头用双面胶固定在左上臂心肺二经上的4个待检测部位，使用配套的Perisoft计算机软件同步观察和储存血流曲线图，并根据探头探测到的血细胞移动速率和其分布状态，利用Perisoft软件分析相关检测部位的微循环血流灌注量。每组受试者均持续检测5min。

(3) 检测部位：心经神门和少海、肺经太渊和尺泽，进行心肺两经对比。

**5.5.5 热传输特性检测**

(1) 检测设备：NEC R450红外热成像仪(日本NEC AVIO公司生产)

(2) 检测过程：受试者进入检测室，仰卧休息15 min，随后开始正式检测。调整红外热像仪摄像头的高度和角度，使受试者左上臂心肺二经上的待检测部位处于摄像头屏幕正中，当大小适中时开始采集图像。通过配套的InfRec Analyzer NS9500计算机软件对红外热像图进行储像、存盘，并分析和提取相关检测部位的温度值数据。每组受试者均持续检测5min。

(3) 检测部位：心经神门和少海、肺经太渊和尺泽，进行心肺两经对比。

**5.5.6** **代谢特性检测**

(1) 检测设备：INVOS 5100C四通道近红外光谱仪(美国Somanetics公司生产)。

(2) 检测过程：受试者进入检测室，仰卧休息15 min，随后开始正式检测。皮肤常规消毒后，撕开探头上的透明胶，将4个探头用双面胶固定在左上臂心肺二经上的4个待检测部位，检测局部氧饱和度(rSO_2_)。每组受试者均持续检测5min。

(3) 检测部位：心经神门和少海、肺经太渊和尺泽，进行心肺两经对比。

**6. 观察指标**

**6.1 微循环特性指标**

包括血流曲线、血流灌注量。

**6.2 热传输特性指标**

包括红外热像图谱、对应部位温度值。

**6.3 能量代谢特性指标**

即局部氧饱和度(rSO_2_)。

## 四、不良事件的处理

### 1．不良事件的类型

本研究主要是采用仪器对患者进行检测，不进行额外的干预，一般不会出现不良事件。

### 2．严重性

医生可以使用以下定义判断所有不良事件以及严重不良事件的严重性。

1. 轻度：不良事件为一过性，患者容易忍受。
2. 中度：不良事件使受试者感到不适，并妨碍受试者的正常活动。
3. 重度：不良事件对受试者的日常活动造成了相当程度的影响，可能会造成功能丧失或危及生命。

### 不良事件的识别、记录和处理

对不良事件的识别，主要通过受试者随时自觉地反馈。出现不良事件时，应将不良事件出现时间及消失时间，不良事件的严重程度与结果，对不良事件采取措施与否及其具体内容等，均要作详细记录。

不良事件发生时，立即由研究者查看病情并判断是否需要进行必要的检查及治疗，对发生的严重不良事件须立刻中断试验，及时迅速而妥善采取解决措施。

### 4．严重不良事件的报告

在出现严重不良事件的情况时，无论该事件是否与仪器检测有关，医生应立即对受试者停止研究，立即进行应急处理和抢救，并在24小时内上报研究中心。

## 五、伦理学和质量

本研究开始前将事先获得伦理委员会批准。在入选患者之前一定要获得其授权同意使用和/或透露个人和/或健康数据。为了保护病人的隐私，病人的年龄会记录在病例报告表上而不记录病人的出生年月，姓名首字母缩写将会记录在病例报告表上。

## 六、数据管理

研究者需根据研究方案要求把收集的数据填入病例报告表。研究结束时，研究者将向数据管理中心递交所有本研究入选的病人的病例报告表，这些病例报告表应是完整的并署名的。从各研究中心收集的病例报告表数据的一致性将被检查，会对不一致的数据发疑问表，需要研究者来澄清。

## 七、统计分析

### 1．统计软件

由不参与前期试验的第三方统计者进行数据统计分析，用统计软件SPSS进行统计分析。

### 2．数据描述

计量资料以均数±标准差(*x*±s)、中位数、最大值、最小值、四分位数描述，计数资料以百分率(%)表示。

### 3．数据统计

所有假设检验均为双侧检验，*P*＜0.05认为差异有统计学意义。对基线数据进行组间可比性评价，在α=5%水平上进行双尾统计学检验。计数资料组间比较采用卡方检验或Fisher精确概率法，计量数据组间比较采用t检验，非参数变量的组间比较采用秩和检验。

### 4．统计分析计划

由专业统计人员完成。在所有数据录入、审核完毕后，统计人员应及时完成统计分析工作，并出具书面统计分析报告。

## 八、最终报告和发表

研究结束后，由主要研究者牵头与各家研究中心的研究者合作编写研究总结报告，各家研究中心的研究者均会在研究总结报告中签字。研究报告中将包括研究目的描述、研究中所使用的方法以及结果和结论。

## 九、质量控制

(1) 由课题组制定统一的检测SOP。

(2) 临床试验正式启动前一个月课题组举行专门的培训会，对所有参与课题的研究者进行统一培训。主要对课题实施方案及各项标准操作规程(SOP)进行重点培训，使每个临床研究人员熟悉掌握研究过程和具体实施细则，保证临床研究结论的可靠性。

(3) 临床研究中所有观察结果都应加以核实，反复确认，以保证数据的可靠性、原始性，确保临床研究中的各项结果及结论均来源于原始数据。

(4) 采用专门人员进行试验数据的收集及统计，以控制试验偏倚。委托专业的数据管理公司进行临床数据管理。

(5) 严格实施每月1次的临床研究质量检查。

## 十、参考文献

[1] Jr F T , Fihn S D , Gibbons R J , et al. 2007 chronic angina focused update of the ACC/AHA 2002 guidelines for the management of patients with chronic stable angina: a report of the American College of Cardiology/American Heart Association Task Force on Practice Guidelines Writing Group to develop the focused update of the 2002 guidelines for the management of patients with chronic stable angina[J]. Journal of the American College of Cardiology, 2007, 50(23):2264-2274.

[2] 中华医学会心血管病学分会. 慢性稳定性心绞痛诊断与治疗指南[J]. 中华心血管病杂志, 2007, 35(3):195-206.

[3] 慢性阻塞性肺疾病诊治指南(2013年修订版)[J].中国医学前沿杂志(电子版),2014,6(02):67-80.

[4] 王淑友. 运用激光多普勒血流成像仪探讨针灸作用原理的初步研究[J]. 中国针灸, 2004, 24(7):499-502.

[5] 针刺对人体体表循经红外辐射轨迹的影响[J]. 针刺研究, 2002, 27(4):255-258.

[6] Raith W, Pichler G, Sapetschnig I, et al. Near-Infrared Spectroscopy for Objectifying Cerebral Effects of Laser Acupuncture in Term and Preterm Neonates[J]. Evidence-Based Complementray and Alternative Medicine,2013,(2013-5-14), 2013, 2013(3):346852.
