# Supplementary material for: The specificity for the correlation between viscera and somato in chronic stable angina pectoris patients and healthy controls: An assessor-blinded and comparative trial
Source: PLoS One. 2025 Sep 26;20(9):e0331868. doi: 10.1371/journal.pone.0331868 (PMC12469238; doi:10.1371/journal.pone.0331868)
Supplement: S3 File — (DOCX) [file pone.0331868.s003.docx]

**Trial study protocol**

**Project Name:** Biological characteristics of meridian phenomena along meridians of heart and lung

**Responsible person:** Jianqiao Fang

**Team Leader Unit:** Zhejiang Chinese Medical University

### I Research background

## Meridian theory, as one of the core contents of basic theories of TCM, guides almost all clinical practices of TCM, including acupuncture and moxibustion. The research on meridian theory is of great significance both in theory and in application.

## Meridian research in China originated in the 1950s and 1960s, mainly exploring the essence of meridians. In the 1970s, the research on the propagated sensation along meridians reached its climax, turning from the search for tissue characteristics to the observation of transmission characteristics along meridians. Many research institutions in China took the lead in investigating the propagated sensation along meridians, and initially confirmed the objective existence of meridian phenomenon. In the mid-1980s, physical methods such as sound, light, electricity, and heat were used to detect meridians, further clarifying the physical and chemical characteristics of the running routes of meridians. Since the 1990s, multi-angle and multi-level research has been conducted, and it was found that Ca2+ and other ions and neurotransmitters along meridians were higher than those along non-meridians. There is specific relationship between meridians and zang-fu organs. With the development of meridian research, many meridian hypotheses have been proposed, such as neurohumoral regulation hypothesis, vascular lymphatic vessel hypothesis, double reflex hypothesis, the third balance system hypothesis and low flow resistance channel hypothesis. However, these hypotheses need to be further improved and confirmed, and it is still far from truly clarifying the essence of meridians.

## Some achievements have also been made in meridian research by foreign counterparts. For example, Japan has carried out a large number of studies on sensation transmission along meridians. South Korea attempted to explore the essence of meridians and collaterals and proposed the concept of primitive piping system. France was the first to use infrared thermography to study meridians and collaterals, and magnetic detection technology was applied to reveal some magnetic properties of meridians and collaterals. The former soviet union applied polarized light detection technology to study the optical characteristics of meridians: Three-dimensional imaging of meridians and collaterals was creatively performed in the UK using magnetic detection electrical impedance tomography.

## Although important progress has been made in the research on meridians and collaterals both in China and abroad, no major breakthrough has been made. The meridian essence is still unclear, and the research results are not enough to guide clinical acupuncture. Meridian research has such problems as disconnection between structural (material basis) research and functional research, and disconnection between basic research and clinical research. Research results of meridian phenomena such as sensation transmission along meridians contain more subjective elements, and lack of scientific technology and means to present meridian phenomena and study their biological characteristics. The research on the correlation between meridians and zang-fu organs is more about the correlation between meridians and acupoints and zang-fu organs, and cannot reflect the relationship and laws between meridians and zang-fu organs well. Besides, it mainly focuses on the correspondence between meridian and viscera, and lacks the comparative study on the correlation between multiple meridians and viscera, as well as the comparison of two or more meridians with the same cross-sectional effect. Therefore, meridian research needs to return to the origin of meridian theory, enhance and innovate research ideas, designs and research methods, and conduct research on the meridian theory, especially the characteristics and mechanisms of meridian-following reaction of the meridian system, and the specific connection phenomena, laws and mechanisms between different parts of the meridian, which will better clarify the key common problems of the meridian theory, improve the meridian theory, and better serve traditional Chinese medicine, especially acupuncture and moxibustion clinical practice.

Based on the above, this project focuses on the core of "meridians reflect the connection between different parts of the body". Starting from the behavior of meridians along the heart and lung meridians, and with the help of three modern science and technology such as laser doppler, infrared thermal imaging and near-infrared spectroscopy, it detects and explores the biological characteristics of meridian responses. Meanwhile, the specificity of the two meridians of heart and lung was studied, as well as the connection between different parts of the body reflected by the meridians and collaterals, as well as their connection characteristics and laws. The results of this study will provide a new scientific explanation for the empirical facts and laws in the meridian theory that are still of great value in clinical practice to date, and are of great significance for the reconstruction of today's meridian theory, as well as acupuncture theory, and the development of acupuncture and moxibustion science.

## II Objectives of the study

(1) To explore the differences in biological characteristics of meridian phenomena along meridians between healthy subjects and patients with cardiopulmonary diseases under physiological and pathological conditions, as well as the relative specificity of the connection between the two meridians of heart and lung and their viscera.

(2) To establish standardized detection techniques and protocols for the biological characteristics of the meridian phenomenon along the meridians of both heart and lung, so as to lay a scientific foundation for the reconstruction of meridian theory and acupuncture theory.

## III Research programme

### 1 Subject selection criteria

Subjects in this study included the following 2 populations: patients with stable angina pectoris (SAP) and healthy subjects.

**1.1 SAP diagnostic criteria**

Refer to the 2007 CHRONIC ANGINA FOCUSED UPDATE of ThE ACC/by the American College of Cardiology/American Heart Association (ACC/AHA) in 2007. The diagnostic criteria for stable angina pectoris refer to those in AHA 2002 Guidelines for the Management of Patients with Chronic Stable Angina [1] and Guidelines for Diagnosis and Treatment of Chronic Stable Angina Pectoris [2] issued by Cardiovascular Branch Association of Chinese Medical Association in 2007.

1. Pain site: The typical angina pectoris site is located behind the sternum or in the left anterior chest, and the scope is often not limited. It can be radiated to the neck, pharynx, jaw, upper abdomen, shoulder and back, left arm and the medial side of left hand finger, or to other sites. Angina can also occur outside the chest, such as upper abdomen, pharynx, and neck. The site of each attack of angina pectoris is often similar;
2. Nature of pain: It usually shows the symptoms of tightness, strangulation, compression, burning, chest stuffiness, chest tightness or the feelings of suffocation and heaviness. Some patients only describe it as chest discomfort, and the subjective feelings vary greatly from individual to individual, but generally it will not be acupuncture-like pain.
3. Duration: paroxysmal, lasting several minutes, usually no more than 10 minutes, nor transient or lasting several hours;
4. Inducing factors and relieving methods: The onset of chronic stable angina pectoris is related to labor force or emotional excitement, such as inducing when walking fast or climbing hills, and relieving after stopping for rest, which usually occurs at the time of labor rather than after. Sublingual nitroglycerin can quickly relieve symptoms in 2-5 minutes;
5. There were no significant changes in the degree, frequency, nature and precipitating factors of angina pectoris within a few weeks.
6. Other examinations support the diagnosis, such as laboratory tests, electrocardiograms, echocardiography, coronary angiography, etc.

### 1.2 Inclusion criteria

**1.2.1 SAP patient inclusion criteria**

(1) Patients who met the diagnostic criteria for coronary heart disease in western medicine (any of the following criteria): ① patients with a clear history of old myocardial infarction, or history of percutaneous coronary intervention (PCI), or coronary artery bypass grafting; ② The results of coronary angiography or coronary CTA indicate that at least one coronary artery is stenotic and the stenosis is ≥50%; ③ Patients whose stress radionuclide myocardial scanning examination showed myocardial ischemia of coronary heart disease; ④ Positive treadmill ECG (male patients only).

(2) Patients with stable angina pectoris who met the diagnostic criteria of western medicine for stable angina pectoris and the Canadian Cardiovascular Society (CCS) classification of angina pectoris severity II-III (see Appendix 1 for relevant classification criteria);

(3) Has a history of angina pectoris of three months or more, and has had no less than two episodes per week in the past one month;

(4) 20 years old ≤ age ≤75 years old, no gender limitation;

(5) clear consciousness, can complete the normal communication;

(6) Those who understood and were willing to obey the research protocol, and signed the informed consent form at the same time.

Note: Patients who met the above six criteria at the same time could be included in this study.

**1.2.2 Inclusion criteria of healthy volunteers**

(1) Healthy subjects who can provide a medical examination report within nearly three months and have a routine physical examination by the researcher to confirm that they have no heart and lung diseases or serious basic diseases such as digestive diseases, urinary diseases, blood diseases, endocrine diseases, nervous system diseases and so on;

(2) Age matched with that of SAP group (no gender limitation);

(3) Those with clear consciousness and ability to complete normal communication;

(4) Those who understood and were willing to obey the research protocol, and signed informed consent form at the same time.

Note: Patients who met the above four criteria at the same time could be included in this study.

**1.3 Exclusions**

**1.3.1 SAP exclusion criteria**

(1) Patients with acute coronary syndrome (including acute myocardial infarction and unstable angina pectoris), severe arrhythmia (such as severe atrioventricular block, ventricular tachycardia, supraventricular tachycardia affecting hemodynamics, and frequent premature beats, especially ventricular premature beats);

(2) Patients with chest pain caused by valvular heart disease, hypertrophic cardiomyopathy, dilated cardiomyopathy, etc.;

(3) Patients with chest pain caused by non-cardiac diseases (such as severe neurosis, climacteric syndrome, cervical spondylosis and chest pain caused by esophagus/lung/chest wall lesions);

(4) patients with pulmonary disease;

(5) Patients with severe basic diseases of digestive system, urinary system, blood system, nervous system and other diseases that cannot be effectively controlled currently;

(6) Patients with mental illness, severe depression, alcohol dependence or drug abuse;

(7) Pregnant or lactating patients;

(8) Patients currently participating in other clinical trials;

Note: Anyone who met any of the above conditions would be excluded.

**1.3.2 Exclusions of healthy volunteers**

(1) Subjects with sudden onset of cardiovascular and cerebrovascular diseases, respiratory diseases, liver diseases, kidney diseases, urinary diseases, hematopoietic diseases and other major diseases during the study;

(2) Patients with mental illness, severe depression, alcohol dependence or drug abuse;

(3) Subjects who were pregnant or nursing;

(4) Subjects currently participating in other clinical trials;

Note: Anyone who met any of the above conditions would be excluded.

### 1.4 **Exclusion and Dropout Criteria**

(1) Cases that failed to meet the inclusion criteria and were mistakenly included;

(2) Subjects after enrollment had poor compliance, failed to comply with the test requirements, and failed to cooperate with the test subjects;

(3) Withdrawal voluntarily due to other personal reasons during the research period;

(4) Patients with acute exacerbation or other critical illnesses requiring rescue measures or hospitalization during the study;

(5) Subjects who experienced serious adverse events or complications and were discontinued from the study because they could not continue to receive the study.

**1.5 Handling of Excluded and Dropped Cases**

After the subject falls off, the researcher shall try to contact the subject by door or telephone and record the reason for the fall off, the time of the last test and all completed test data. In the case of dropping out of the trial due to adverse reaction or acute exacerbation of the disease, the investigator shall take corresponding treatment measures according to the actual situation of the subject. The original medical records of all patients who were excluded and dropped out should be retained for future reference.

### 2. Subject Enrollment

This clinical trial is expected to include 80 subjects, 40 patients with SAP and 40 healthy subjects.

1. **Subject Source**

SAP patients were mainly recruited from the relevant outpatient and ward of the Third Affiliated Hospital of Zhejiang University of Chinese Medicine. Healthy subjects were mainly recruited from the physical examination center of the Third Affiliated Hospital of Zhejiang University of Chinese Medicine.

1. **Research Start and End Dates**

Estimated period: January 2020 to January 2021

1. **Implementation of the study**

**5.1 Sample Size Estimation**

This project is an observation and study on the biological characteristics of meridian phenomena detected by a variety of modern science and technology. Compared with common clinical trials, there is no uniform standard for the estimation of sample size. Based on the similar meridian biological characteristics detection experiments in China and abroad [4–6], and the actual conditions of this study, two groups of subjects were proposed to be included. There were 40 patients in the SAP group and 40 volunteers in the health group, totaling 80 subjects.

### 5.2 Subject Entry

Prior to initiating the study, the investigator should provide the subject or his/her designee with a clear, colloquial, detailed explanation of the purpose, content, and potential risks and benefits of the study, and the subject or his/her designee and investigator should sign and date an informed consent form. Subjects or their designees must have signed informed consent before they can be screened and subsequently enrolled in the study.

Subjects with written informed consent were required to undergo the following evaluations, which were recorded, and to determine whether a subject could be enrolled in the study based on inclusion and exclusion criteria.

1. Demographic and disease data: Demographic data included gender, name, age, occupation, height, and weight of the subjects; disease data included disease diagnosis, current medical history, past medical history, disease classification, and current medication intake.
2. Vital signs: including temperature, heart rate, respiration and blood pressure;
3. Laboratory tests: routine blood test and biochemical tests (including AST, ALT, BUN, Cr, GLU and blood lipid);
4. Electrocardiogram.

### 5.3 Grouping

Eighty subjects (40 cases in each group) were included in the SAP group and the healthy group.

### 5.4 Blind Method

During the implementation of this study, subjects and those who performed indicator detection and entry were not blinded. In the data summarizing stage, blind method was used for statistical analysis, and the third-party statisticians who did not participate in the preliminary experiment conducted the subsequent statistical analysis of data.

### 5.5 Detection Scheme

Laser doppler, infrared thermal imaging and near infrared spectroscopy were used to detect the microcirculation, heat transfer and metabolism characteristics of the cardiopulmonary bypass in the physiological and pathological states.

**5.5.1 Acupoint positioning**

The acupoints were positioned according to the national standard of Nomenclature and Location of Acupuncture Points (GB/T 12346-2006).

Taiyuan (LU9): Located in the anterior region of wrist, between the styloid process of radius and scaphoid, and in the ulnar depression of the abductor pollicis longus tendon.

Chize (LU5): It is located in the transverse crease of the elbow, in the depression on the radial side of the tendon of biceps tendon.

Shenmen (HT7): It is located in the anterior region of the wrist, at the ulnar end of the transverse crease on the vola carpi, and at the radial depression of the flexor carpi ulnaris tendon.

Shaohai (HT3): It is located in the anterior region of elbow, with transverse horizontal crease of elbow and leading edge of medial epicondylar of humerus.

**5.5.2 Detection precautions**

(1) Patients in the SAP group maintained their current regimen throughout the study cycle. During this period, if new drugs or other treatments were added for various reasons, the explanation must be timely given to the investigator, and the investigator shall make a detailed record. The subjects in the health group were guaranteed not to take any medicine during the study. If medicines and other treatments were used for sudden illness, the records would also be recorded by the researcher and whether to exclude them would be evaluated.

(2) All subjects were instructed to prohibit drinking tea, alcohol and coffee as well as smoking within two days prior to the test. No exercise or food for 1 hour prior to testing.

(3) The subjects were asked to keep quiet and natural breathing throughout the test, and to avoid limb movement as much as possible.

**5.5.3 Detection environment**

A special detection chamber was set up with the room temperature controlled at (25 1) C and the relative humidity controlled at 40%-50%. Indoor no direct sunlight and obvious air convection.

**5.5.4 Detection of microcirculation characteristics**

(1) Detection equipment: PeriFlux System 5000 model four-channel laser Doppler blood flow meter (manufactured by PeriMed, Sweden)

(2) Detection process: The subjects entered the detection room and rested in the supine position for 15 min. Then the formal detection was started. Four probes were fixed on the four parts to be detected on the cardiopulmonary bypass of the left upper arm with double-sided adhesive tape, and the matching Perisoft computer software was used to synchronously observe and store the blood flow curves. According to the blood cell movement rate and its distribution state detected by the probes, the Perisoft software was used to analyze the microcirculatory blood perfusion in the relevant detection parts. The test was continuously performed for 5min in each group.

(3) Detection sites: Shenmen (HT 7) and Shaohai (HT 7) in the heart meridian, Taiyuan (SP 6) and Chize (SP 9) in the lung meridian were compared between the cardiopulmonary meridians.

**5.5.5 Detection of heat transfer characteristics**

(1) Detection equipment: NEC R450 infrared thermal imager (manufactured by NEC AVIO, Japan)

(2) Detection process: The subjects entered the detection room and rested in the supine position for 15 min. Then the formal detection was started. The height and angle of the camera head of the thermal infrared imager were adjusted so that the part to be detected on the cardiopulmonary bypass of the left upper arm of the subject was in the middle of the camera screen, and image collection was started when the size was moderate. The infrared thermography was stored and archived with the matching InfRec Analyzer NS9500 computer software, and the temperature data of the relevant detection parts were analyzed and extracted. The test was continuously performed for 5min in each group.

(3) Detection sites: Shenmen (HT 7) and Shaohai (HT 7) in the heart meridian, Taiyuan (SP 6) and Chize (SP 9) in the lung meridian were compared between the cardiopulmonary meridians.

**5.5.6 Detection of metabolic characteristics**

(1) Detection equipment: INVOS 5100C four-channel near-infrared spectrometer (manufactured by Somanetics, the US).

(2) Detection process: The subjects entered the detection room and rested in the supine position for 15 min. Then the formal detection was started. After routine skin disinfection, the transparent adhesive was torn off from the probes, and four probes were fixed on the four parts to be tested on the cardiopulmonary bypass of the left upper arm with double-sided adhesive tape, to detect local oxygen saturation (rSO2). The test was continuously performed for 5min in each group.

(3) Detection sites: Shenmen (HT 7) and Shaohai (HT 7) in the heart meridian, Taiyuan (SP 6) and Chize (SP 9) in the lung meridian were compared between the cardiopulmonary meridians.

**6. Outcome measurements**

**6.1 Microcirculation characteristic outcomes:** the perfusion unit (PU)

**6.2 Heat transfer characteristic outcomes**

Comprise an infrared thermal image and a temperature value of a corresponding part.

**6.3 Metabolism characteristic outcomes:** regional oxygen saturation (rSO2)

## IV. Treatment of adverse events

### 1. Types of adverse events

In this study, the patients were detected using instruments without additional intervention, and generally no adverse event occurred.

### 2. Severity of all adverse events

Physicians can use the following definitions to determine the severity of all adverse events and serious adverse events.

1. Mild: Adverse events are transient and easily tolerated by patients.
2. Moderate: The adverse event caused subject discomfort and impaired normal subject activity.
3. Severe: Adverse events that have a significant impact on subjects' daily activities may result in loss of function or be life-threatening.

### Identification, recording and handling of adverse events

Adverse events were identified mainly through voluntary feedback from subjects at any time. When an adverse event occurs, the occurrence time and disappearance time of the adverse event, the severity and result of the adverse event, and whether to take measures for the adverse event and the specific content shall be recorded in detail.

When an adverse event occurs, the investigator shall immediately review the condition and determine whether necessary examination and treatment are required. The trial shall be immediately interrupted for serious adverse events, and timely, rapid and appropriate measures shall be taken to solve the problem.

### 4. Report of serious adverse events

In the case of serious adverse events, regardless of whether the event was associated with an instrument test, the physician should immediately stop the study for the subject, provide immediate emergency management and salvage, and report to the study center within 24 hours.

## V. Ethical considerations

Approval from the Ethics Committee will be obtained prior to the start of the study. Patients must be authorized to use and/or disclose personal and/or health data prior to enrollment. To protect the privacy of the patient, the patient's age is recorded on the case report form without the patient's date of birth and the initials are recorded on the case report form.

## VI. Data management

The investigator will fill in the collected data on the case report form as required by the protocol. At the end of the study, the investigator will submit to the data management center case report forms for all patients enrolled in this study, which should be complete and signed. The consistency of the case report form data collected from the study centers will be checked and inconsistent data will be questioned by the form and will need to be clarified by the investigator.

## VII. Statistical analysis

### 1. Statistical software

Third-party statisticians who did not participate in the preliminary tests were used for statistical analysis, and SPSS statistical software was used for statistical analysis.

### 2. Data Description

Measurement data were described as mean standard deviation (x̄ s), median, maximum, minimum, and quartile, and enumeration data were expressed as percentage (%).

### 3. Data statistics

All hypothesis tests were bilateral tests, and the difference was considered to be statistically significant (P < 0.05). Inter-group comparability was evaluated for baseline data and a two-tailed statistical test was performed at α=5%. The chi-square test or Fisher's exact probability method was used for comparison among enumeration data groups, the t test was used for comparison among measurement data groups, and the rank-sum test was used for comparison among non-parametric variables.

### 4. Statistical analysis plan

Done by professional statisticians. In all data entry, after the audit, the statistical personnel should be timely completion of statistical analysis, and issue a written statistical analysis report.

## VIII. Final report and publication

At the end of the study, a study summary report was prepared under the leadership of the principal investigator in collaboration with the researchers from each study center, and the researchers from each study center signed the study summary report. A description of the purpose of the study, the methods used in the study, and the results and conclusions will be included in the study report.

## IX. Quality control

(1) The research group shall formulate unified testing SOP.

(2) A month before the formal start of clinical trials, the research group held a special training meeting to uniformly train all the researchers participating in the study. We mainly focused on training the implementation plan of the project and various standard operating procedures (SOPs), so that each clinical researcher was familiar with the research process and the specific implementation details, and to ensure the reliability of clinical research conclusions.

(3) All observations in clinical studies should be verified and repeatedly confirmed to ensure the reliability and originality of data and that all results and conclusions in clinical studies are derived from original data.

(4) Trial data were collected and counted by special personnel to control trial bias. Clinical data management was entrusted to a professional data management company.

(5) Strictly carry out the clinical study quality inspection once a month.

## X. References

[1] Jr F T , Fihn S D , Gibbons R J , et al. 2007 chronic angina focused update of the ACC/ AHA 2002 guidelines for the management of patients with chronic stable angina: a report of the American College of Cardiology/ American Heart Association Task Force on Practice Guidelines Writing Group to develop the focused update of the 2002 guidelines for the management of p atients with chronic stable angina[J]. Journal of the American College of Cardiology, 2007, 50(23):2264-2274.

Cardiovascular Disease Association of Chinese Medical Association. Guidelines for the diagnosis and treatment of chronic stable angina pectoris [J]. chinese journal of cardiology, 2007,35 (3): 195-206.

[3] Guidelines for the diagnosis and treatment of chronic obstructive pulmonary disease (revised edition in 2013) [J]. China Journal of Advanced Medical Sciences (electronic edition), 2014,6(02):67-80.

WANG Shu-you. Preliminary study on the mechanism of acupuncture using laser Doppler blood flow imaging [J]. China Acupuncture and Moxibustion, 2004, 24(7):499-502.

Effect of acupuncture on infrared radiation track along meridian of human body surface [J]. acupuncture research, 2002, 27(4):255-258.

[6] Raith W, Pichler G, Sapetschnig I, et al. Near-Infrared Spectroscopy for Objectifying Cerebral Effects of Laser Acupuncture in Term and Preterm Neonates[J] . Evidence-Based Complementray and Alternative Medicine,2013,(2013-5-14), 2013, 2013(3):346852.
